# Family Planning and the Samburu: A Qualitative Study Exploring the Thoughts of Men on a Population Health and Environment Programme in Rural Kenya

**DOI:** 10.3390/ijerph14050528

**Published:** 2017-05-13

**Authors:** Loren Kock, Audrey Prost

**Affiliations:** Institute for Global Health, University College London, London WC1N 1EH, UK; audrey.prost@ucl.ac.uk

**Keywords:** Kenya, family planning, maternal health, environmental health, gender, natural resources, climate change, conservation

## Abstract

Population Health and Environment (PHE) strategies are argued to improve ecosystem and human health by addressing family size and its effects on natural resource use, food security, and reproductive health. This study investigates men’s views on a PHE family planning (FP) programme delivered among the pastoral Samburu tribe in rural northern Kenya. Three focus group discussions and nine semi-structured interviews were conducted with 27 Samburu men. These discussions revealed support for environmentally-sensitised family planning promotion. Men highlighted their dependency on natural resources and challenges faced in providing for large families and maintaining livestock during drought. These practices were said to lead to natural resource exhaustion, environmental degradation, and wildlife dispersal, undermining key economic benefits of environmental and wildlife conservation. Relating family size to the environment is a compelling strategy to improve support for FP among Samburu men. Kenyan policy-makers should consider integrating community-based PHE strategies among underserved pastoral groups living in fragile ecosystems.

## 1. Introduction

### 1.1. Population Health and Environment Programmes: Principals and Evidence of Impact

Population Health and Environment (PHE) programmes are cross-sectoral development initiatives that integrate environmental conservation, health, and family planning (FP) interventions. Population growth is a key factor driving environmental degradation, which itself has negative impacts for local populations by undermining household economies and food security through land exhaustion and drought [1,2]. PHE strategies address natural resource use, food security, and reproductive health as connected issues all affected by family size: by reducing population pressures on the environment, ecosystem and human health are improved [3,4]. An evaluation of World Wild Fund for Nature-sponsored PHE initiatives worldwide concluded that to create demand for FP and environmental conservation, programmes must demonstrate improvements in the livelihoods of local populations [5].

In Saadani National Park, Tanzania, a situational analysis revealed that among 60 households spread between six villages, 33% of respondents thought that food insecurity was linked to high human population and low numbers of fish, which was their main source of income. Seventy-nine percent (79%) believed that absence of FP could cause resource scarceness in the future [6].

Combining health and environmental messages has the potential to reach a wider audience than single sector interventions, improving men’s support for FP [7,8]. The organisation Blue Ventures in Velondriake, coastal Madagascar, reported that after linking FP and family size with marine environmental resource management and food security, men became more engaged in reproductive health issues [3,9]. The Ethiopian Guraghe People’s Self Help Development Organisation report that PHE programmes develop greater male acceptance and involvement in FP [8]. In PHE sites, 30.2% of men supported contraceptive use compared to 7.3% in Reproductive Health only sites, a statistically significant difference. The United States Agency for International Development’s (USAID) Rwandan integrated community health programme for coffee farm workers, found that educating the community about FP use led to greater understanding of the important economic benefits of FP, ultimately contributing to a favourable change in men’s attitudes towards FP [10].

Evidence for the benefits of PHE programmes to improve FP outcomes is found in numerous contexts. In 23 of the 35 PHE projects reviewed, increases in FP use were reported [8]. In Blue Ventures’ Malagasy PHE project, integrating health services with conservation activities provided an opportunity to communicate resource management issues such as over-fishing with women from outside the marine conservation area who visited the reproductive health clinics [3]. Furthermore, a significant increase was observed in sexually active women using contraception, from 25% in 2009 to 59% in 2013, along with the general fertility rate decreasing by 28% over the same period [11]. Robson and Rakotozafy estimated that contraceptive provision in this Malagasy community prevented 800 unwanted pregnancies since 2007 [12]. Harris et al., estimated that such provision prevented 88 unsafe abortions in the community between 2007 and 2010, with couple-years of protection (CYP) rising from 39.5 to 464 over the same period [9]. Furthermore, among WWF-sponsored PHE projects in Asia and Africa, contraceptive prevalence rate (CPR) increased considerably in Kenya’s Kiunga district, Madagascar’s Spiny Forest, Nepal’s Khata region, and in Roxas district, Philippines [5,13]. Results also indicated long-term effects of PHE FP programmes on fertility and reproductive health, showing a decline in birth rates.

A quasi-experimental design was used in Palawan, the Philippines to test whether an integrated coastal resource management and reproductive health (CRM + RH) PHE programme would deliver significant improvements in both CRM and RH outcomes compared to single sector interventions that focused on CRM or RH alone [14]. Between 2001 and 2007, the use of contraceptives in young men and women living in the CRM + RH group increased more than in the RH only group. The CRM + RH programme showed significantly greater impact on five out of nine food security and reproductive health indicators, while performing to the same standard with the remaining indicators. A 2011 retrospective cohort study, which randomly selected 34 PHE-integrated villages and 18 non-PHE villages in the Visayas region of the Philippines, found a statistically significant difference where women from community-PHE programme sites had greater knowledge about contraceptives than those in non-PHE control villages [15].

In a comparative cross-sectional study conducted by Sinaga et al., in Southern Ethiopia, the CPR of a PHE study group was 78% compared with 52% in the non-PHE group, a statistically significant difference [16]. The authors of this study acknowledge that the CPRs measured were very high for the Ethiopian context, perhaps due to the local health authority using a national holiday to distribute family planning methods. However, this does not discount the significant difference in CPR between the PHE and non-PHE groups. Furthermore, women in the PHE-group were four times more likely to use contraceptives compared with women in the non-PHE group. The study also indicated that women with husbands who supported FP utilization were 17 times more likely to use contraceptives.

Limited evidence exists on effective methods to increase male involvement in FP in Sub-Saharan Africa (SSA), and few studies have sought to specifically understand male perspectives on FP through PHE programmes; none have done so in the Samburu context. It is therefore important to investigate perspectives in the Samburu community in Kenya. More research is needed on the social conditions and attitudes that shape Samburu male fertility ideals, which may be more dynamic than the cultural assumptions that are made about their fixed position.

### 1.2. Kenya: Family Planning and Population Health

The Kenyan government has implemented several FP and reproductive health strategies including the 2007 National Reproductive Health Policy, the National Reproductive Health Strategy (2009–2015), the Population Policy for National Development (2012–2030), and the Costed Implementation Plan for Family Planning (2012–2016). While these initiatives have contributed to increases in the national contraceptive prevalence rate (CPR) (27% in 1989 up to 58% in 2014 Demographic and Health Survey (DHS)), changes in health system governance have complicated service delivery at the local level. The national health system was decentralised in 2013, moving healthcare decision-making and delivery to the county level. Maintaining focus on FP during decentralisation has been difficult owing to competing county health priorities: only six out of 47 counties had FP budgets in 2015 [17,18]. FP service provision therefore remains at the national level, with non-governmental organisations (NGOs) acting in contexts where it is lacking.

The European Union and Amref Health Africa, in partnership with the Kenyan Ministry of Health and a local NGO, have implemented a four-year project in Samburu County to strengthen community health systems by addressing social disparities in maternal, newborn and child health, nutrition, and family planning [19]. The lack of health services, poor road access, and remoteness of Samburu County remain major challenges in the supply of health services to its population.

The Community Health Africa Trust (CHAT) is a local NGO that delivers mobile health clinics to remote and underserved communities in Kenya. The pastoral Samburu tribe live in and around Samburu County in northern Kenya, and have a population of approximately 150,000 [20]. This region experiences frequent droughts, limiting water and food availability. In addition to environmental pressures, the traditional livelihoods of East African pastoral societies are being squeezed politically and economically through loss of herding land, changing land use, out-migration, and the commodification of livestock [21]. Using provision of medical care as an entry point into the Samburu community, CHAT implements a holistic PHE FP initiative which provides contraceptives and seeks to communicate the links between family size and the environment. CHAT’s PHE programme is thought to improve both maternal and child health while reducing population pressures on the environment, and conserving wildlife and other key livestock resources such as water and grazing lands.

Samburu County has one of the highest total fertility rates in Kenya at 6.3 [22] and a maternal mortality rate (MMR) of 472 deaths per 100,000 live births [23]. Other data collected in Samburu estimate that 29% of deliveries are carried out by a skilled birth attendant, 24.5% occur in a health facility, and the contraceptive prevalence rate (modern methods) is 20% [22]. The unmet need for FP has been estimated at 50% [19]. A high rate of population growth along with increased grazing from Samburu livestock in a resource-scarce region result in increased pressures on the environment. Due to culturally constructed gender roles in Samburu society, women are thought to be more vulnerable to environmental hazards such as flooding and drought [24]. Samburu society has been described as a patriarchal gerontocracy. An ethnography carried out by Paul Spencer highlighted that older men hold political and marital influence over women and younger men [25]. Although not universally, older men are known to practice polygyny, marrying women from the age of fifteen and sometimes younger, while circumcised “Moran” usually under the age of thirty are largely prevented from marrying for 15 years and spend much of their time away from the community. Spencer has argued that the desire for large families stems from the need to ensure that family livestock, which are central to the Samburu’s pastoral economy, are maintained. In addition to this, having more children is seen as insurance for the care of elders in the family. Through its PHE initiative, CHAT reports increasing acceptance of FP among men. Their involvement is essential to the success of a family planning programme in this context as in other similar patriarchal contexts.

### 1.3. Men as Barriers to Meeting Unmet Need for FP

A literature review examining reasons for non-use of contraception among women with an unmet need for FP found that 25% of women in Africa state that they, their partner, or a close relation is opposed to the use of contraception [26]. Studies into gender attitudes and fertility aspirations among men in East African countries with high fertility found that women’s fertility preferences are strongly influenced by their husbands’ desire to have many children [27,28]. In West Pokot County, Kenya, men and women highlighted low male involvement in FP as the major obstacle to the uptake of FP services [17]. These findings are supported by research in Western Kenya investigating men’s perspectives on their role in FP. Focus group discussions with 106 married men age 20–66 indicated that their disapproval of contraceptive use was largely due to concerns about losing their male identity and anxiety about infidelity among their wives [29]. An evaluation report of Amref Health’s 2014 “strengthening community health systems” project highlighted resistance towards FP among men in Samburu County [19]. Men feared their wives would never deliver again if they started using contraceptive methods, and this was described as a concern, since having many children was a sign of wealth in the community. These reports recommended greater male education and sensitisation on the importance of FP, arguing that FP programmes which solely focus on women compound the lack of acceptance among men.

While much of the fertility literature importantly involves women, this research investigates one of the key barriers to the uptake of family planning: the role of men in a largely patriarchal Samburu context. The objective of this study was to investigate what Samburu men think about the relationship between family size and the environment, the unique component of CHAT’s FP programme. Findings may be used to influence future implementation of the programme through a greater understanding of the perspectives of Samburu men in the population, health, and environment context.

## 2. Materials and Methods

Purposive quota sampling was used to recruit 27 Samburu men. Nine semi-structured interviews (SSIs) and three focus group discussions (FGDs) were then conducted with these men in May 2016. The SSIs included men who were not involved in the FGDs. Assisting with data collection was a research assistant (a 22-year-old Samburu man from a different part of the County) and two women who work as FPCORPs (Family Planning Community-Own Resource Persons) with CHAT assisting by mobilising men in the community to participate in this research. Being from the local community, these women were trusted, and without their presence the research would not have been possible.

A flexible topic guide (see Supplementary Materials) was used to focus on participants’ thoughts on family planning, family size, and the environment. Broad introductory questions engaged participants with the topics before using more specific questions and probes to gain further insight into their thoughts. FGDs were appropriate for this study because they allow for an exploration of social consensus and the opportunity to debate controversial issues through discussion. Furthermore, while family size and fertility can be argued as a couple/family level issue, the use and sharing of resources (including livestock) is often a community decision and therefore it was important to use focus groups to explore this. All SSIs and FGDs were conducted in the local Samburu language “Maa”.

### 2.1. Study Setting

The research took place in five Samburu pastoral communities in the East of the County. The Samburu face several major barriers to accessing health services. The first is remoteness: villages included in this study were located between 15 and 50 km to the nearest health facility, and the Samburu travel across difficult terrain to access it. Some health services also cost money, and some families were too poor to afford them. To address these barriers, CHAT staff travel by whatever means necessary to deliver health and FP services to these underserved communities, including by car, camelback, or foot. CHAT delivers its FP strategy through the employment of FPCORPs, who help to ensure community acceptance of the programme, considering the existence of traditional and familial opposition to FP.

### 2.2. Study Participants

Twenty-seven men (all previously engaged with CHAT’s PHE programme) were recruited from the Lodungokwe, Lengei, and Lolua communities and the livestock markets at Lengusaka and Lolkuniani. Participants were stratified across three age groups (Table 1) to ensure a wide age range was represented and because during FGDs, men (particularly those aged 18–30) would be more comfortable discussing issues within their own peer group. Had discussions included a mixture of generations, there might have been a risk of younger men’s opinions being strongly influenced by dominating elders. Men age 18–30 represented the “Moran”, the warrior generation that begins at circumcision and lasts for approximately 15 years. During this time, a Moran is unable to marry and often lives away from home, carrying out important security and livestock management duties for his community. Men age 30–45 represented the younger generation of elders who had completed their time as Moran and had married, returning to their homes. The final age category was 45 and over, representing the older generation of elders.

### 2.3. Ethical Considerations

Ethical approval was received from the University College London (UCL) ethics committee (Project ID: 8323/001) and the Kenyan National Commission for Science and Technology (who granted the researcher with a permit to complete this project) and were affiliated with the Kenyan Medical Research Institute. Any personal health concerns of participants would have been passed on to CHAT and the most appropriate health facility, however, no such issues were raised. Data collected were anonymised during transcription and stored on a password-protected computer; all recordings were deleted following transcription.

### 2.4. Participant Recruitment

Upon arrival in Samburu, the lead researcher met with a research assistant and the FPCORPs to discuss participant recruitment. One FPCORP informed men of my research project, mobilising them in advance. A major challenge in recruiting Samburu men was that they were often out grazing their herds of livestock, and therefore unable to take part in potentially time-consuming qualitative research. Therefore, all study sites were purposively chosen according to when men of the required age group were available to take part.

The FP CORPs arranged Lodungokwe village to be the site for the FGD and interviews with men in the 18–30 group, Lolua for the 30–45 FGD, and Lengei for the 45+ FGD. SSIs with men of the 30–45 and 45+ groups were arranged at the Lengusaka and Lolkuniani livestock markets, respectively. All participants were provided with information and consent forms written in English, which were read and translated into Maa for them as necessary. For the men who could not write, consent forms were signed using a thumbprint.

### 2.5. Data Collection

The FGDs at Lodungokwe, Lengei, and Lolua, and the SSIs at Lolkuniani and Lodungokwe took place at a time and place of the participants’ choosing. Because the lead researcher is not fluent in Maa, a research assistant facilitated all SSIs and FGDs. He was selected for this role because he was fluent in English, Maa, and Swahili, had previous research experience, and because being a Samburu himself, he was familiar with the context and issues discussed, and understood the important cultural and emotional meanings in men’s answers. Time was spent discussing the topic guide and how it would be translated with him before commencing data collection; specific emphasis was placed on keeping the questions open, allowing the participants to answer in the same manner. SSI responses were simultaneously translated so that the researcher was able to probe specific areas and make detailed notes. Also present at the SSIs and FGDs were the two FPCORPs. Without their presence, as trusted members of the community, the researcher would not have been able to conduct data collection. Through experience, the Samburu are fearful of exploitation and will not engage with research projects unless there is transparency about the intended outcomes. Because the lead researcher was linked to CHAT, the strong relationship that the NGO has with the communities was fundamental to their willingness to discuss and voice their opinions. Due to a lack of time, back-translation of transcripts was not completed, which would have verified the accuracy of translations.

### 2.6. Analysis

**Figure 1:**
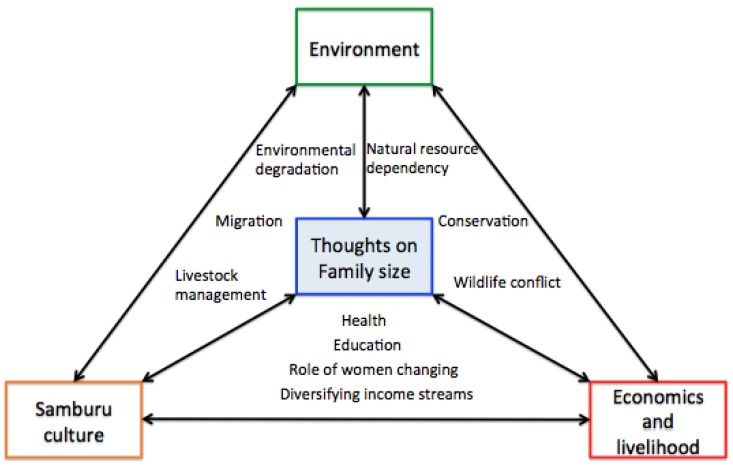
Key themes.

After translation and manual transcription of the data, the transcripts were uploaded onto the qualitative analysis software package NVivo 11. Thematic analysis [30] was used, due to its flexibility as a method. The data was read frequently and carried out progressive low-level coding, which produced 46 codes related the topics investigated. Further thematic analysis generated key themes (Figure 1); a concerted effort was made to check for men who had different opinions to the rest of the cohort, and to place them within the greater framework of themes being developed.

## 3. Results

The lead researcher identified key themes that reflected men’s thoughts of and around CHAT’s family planning programme (Figure 1). It became clear that men’s thoughts on the relationship between family size and the environment could not be described without incorporating themes related to Samburu culture, economics, and livelihood.

Three main themes, described in Figure 1, explained how Samburu men’s thoughts on family size and the environment are closely linked to Samburu culture and factors related to economics and livelihood.

### 3.1. The Environment and Samburu Culture

#### 3.1.1. Migration-Land Exhaustion Cycle

A perception among men was that the traditional Samburu aspiration to have large families was a key factor behind the exhaustion of environmental resources. They universally supported the environmental education that the FP programme delivers.

It is good to educate about FP through the environment and resource availability. If we continue to produce large families we will exhaust the grasses and water we have in Samburu, and be forced to migrate to new places. Our livestock, which we are entirely dependent upon cannot survive on bare land.

SSI-03 (45+)

This migration and land exhaustion cycle was often cited as a common practice among large family groups.

Larger families require the cutting down high numbers trees (sic) to build a settlement. We cut them down, and then when we are faced with a drought, we move and once again cut down more trees to settle. This cycle repeats itself.

SSI-02 (30–45)

#### 3.1.2. Livestock Management

Men acknowledged that much of the environmental degradation and resource exhaustion due to large families was related to the traditional aspiration for, and dependence on, large herds of livestock.

With larger families there are more livestock, so grasslands are exhausted and there is nowhere to feed them.

M6 FGD (18–30)

Reducing livestock numbers was seen as a strategy to better manage available resources.

Families cannot survive without a healthy environment. Reducing families and the size of our livestock herds will slow down land degradation and allow us to better use our resources.

SSI-02 (30–45)

#### 3.1.3. Environmental Change

Older men highlighted how the environment had changed over their lifetime. They said that it was healthier in the past and that their lives were harder today due to frequent drought. Men age 18–30 believed that unpredictable rainfall patterns were a consequence of human activity. Deforestation was mentioned on several occasions in relation to family size.

Large families cause destruction. We have to run to other places to seek pastures, and it is because of our people cutting trees. The trees bring rain. Tree cutting is widespread now and they are not just cutting dry dead trees but green trees too.

M2 FGD (18–30)

#### 3.1.4. Family Size Compromise

The benefits of large families were frequently cited, but most men believed that in the current environmental context, where natural resources were scarce it was better to compromise and support smaller families.

In recent years it has been difficult to care for large families because life has become very hard. When you have four wives and you have 500 shillings, it will not be enough for all of them and our children. Large families are good if you have enough resources to provide for them, but at the moment this is hard. Smaller families are good because you can look after them.

M3 FGD (45+)

Not all men saw reducing their livestock herds as an option, despite acknowledging the effect that large families have on the environment.

Large families lead to practices such as poaching and the exhaustion of the land. But I do not support the reduction in our livestock. Because if we lose our livestock then how will we survive? We depend almost entirely upon them. This land cannot be used for anything else.

SSI-01 (45+)

One elder argued that because of the vast stretches of land available to the Samburu tribe relative to their small numbers, they should continue to populate the region. He therefore opposed the use of FP.

We Samburu are very few but we have a lot of land. Other tribes have filled their regions and they are coming to our land now. Because of this I think that we don’t need FP. If we restrict our size now, while we are still few, what will be our situation in the future? Other tribes who have filled their lands should be using FP, but we should not because we have not filled our lands.

M2 FGD (45+)

### 3.2. The Environment, Economics, and Livelihood

#### 3.2.1. Natural Resource Dependency

All men discussed natural resource dependency, and how their way of life is closely tied to the availability of natural resources.

I depend on the environment. It provides me with resources like timber for building and trees provide shelter. Water and trees are the most important resources. The dried part of trees can be used for firewood and fencing, and the trees bring rain. There is also sand and grass. Sand is used for building houses, grasses feed our livestock.

SSI-02 (18–30)

#### 3.2.2. Environmental Economy

The economic value of the environment was mentioned regularly. Natural resources provide a form of income either directly through sale or indirectly through maintenance of livestock that are then sold at market.

We get lots of benefits from the environment. These acacia trees are food for our livestock during drought. People travel from far away to buy our sand and rocks.

SSI-03 (45+)

Livestock are important, they are our farms. We sell them to buy food and fund other activities.

M6 FGD (30–45)

#### 3.2.3. Conservation

Men highlighted the economic benefits of the community wildlife conservancies coordinated by the northern rangelands trust (NRT), which were said to have increased employment and also brought income and educational grants to the community through increased wildlife tourism. Smaller families were understood to be better for wildlife conservation, which itself has economic value.

We have a good relationship with wildlife, and conservation is a useful practice here. They help educate our children and people in our community are finding employment with the conservancies.

SSI-03 (45+)

#### 3.2.4. Wildlife Conflict

Men highlighted that large families and their livestock herds cannot coexist peacefully with wildlife. Conflict with wildlife remains a constant problem for the pastoral Samburu, and strains their relationship with conservancies.

We have conflict with wildlife when we have larger families. We need to enter the conservancies to look for pastures during drought. Morans have fought with conservancies in the past because they think that they are taking their grazing lands. The conservancies do not want our livestock to enter protected land.

M1 FGD (18–30)

Large families interfere with wildlife and conservation. Wildlife cannot survive in overpopulated places. They are disturbed and attack people and livestock.

M5 FGD (45+)

The economic benefit of conservation is undermined when conflict occurs through wildlife attacking and killing valuable livestock.

There are conflicts with hyenas and wild dogs that attack our goats. I have to attack these animals to protect my livestock. Elephants scare our livestock, so they don’t feed near the elephants. We have to wait for them to leave. If they don’t leave then you have to attack them so that they are scared and run away.

M4 FGD (18–30)

### 3.3. Samburu Culture, Economics, and Livelihood

#### 3.3.1 Culture and Tradition

Men spoke of the traditional Samburu association between family size and wealth, and how because of this, giving birth to boys remains an important cultural aspiration.

With larger families, there is greater continuation of a bloodline. The sixth generation from our birth passed a long time ago but we know their descendants. If a woman does not provide any boys, then a man will have to marry another woman who can provide him with boys so that he can continue his bloodline. When a large family becomes successful, with the children finding employment, then parents benefit.

M5 FGD (45+)

It was frequently stated that a major benefit of having a large family was that family labour could be divided amongst the children.

Larger families are good though because they allow division of labour. Some children can go to school and the others can stay to care for livestock.

M5 FGD (18–30)

However, some men believed that such traditions should change, and that communities in which they are common are poorer.

I don’t think that it is right for people to have large families with many wives now, because it leads to poverty in the community. We should stop practicing polygyny. People shouldn’t have lots of kids and not be able to look after them, that is shameful for a man.

SSI-01 (30–45)

Men thought that there remained many Samburu who held on to traditional desires for large families and multiple wives. Men wished that FP education would reach these communities so that they could learn the environmental and economic benefits of having smaller families.

#### 3.3.2. Education

Participants mentioned that in the past, the Samburu did not value education but that nowadays it is seen as a means to gain employment and better provide for your family.

We now understand that there are changes that we have to make in order to succeed as a community. Now that we have education here, we are able to allow children to benefit from it. In the past we used to think that sending them to school would make them more foolish.

M2 FGD (45+)

Individuals across all age groups believed that having large families conflicted with their desire to educate their children.

If God gives you a very clever child, and you take him/her to school, but you take them out of school because due to having a large family you cannot afford the fees, that is not good.

M2 FGD (45+)

#### 3.3.3. Maternal and Child Health

Men saw health improve among women (and their children) who were able to control and space their births through the use of contraceptives provided by CHAT.

Women who looked old because of giving birth frequently, look young again when she takes contraceptives and stops giving birth. Their children are healthier, since their mothers can focus on looking after one child at a time. There are far fewer or no more cases of mothers dying during childbirth.

M5 FGD (30–45)

The overall consensus was that birth spacing was one of the programme’s benefits, because it allowed mothers to recover after giving birth and focus on caring for their newborn, before giving birth again. However, ceasing to give birth permanently was rarely discussed as the purpose of FP.

When a woman gives birth, using contraceptives allows the child to grow before another is born, and it allows time for a woman’s womb to heal. Women have more energy then to have another child.

M3 FGD (30–45)

#### 3.3.4. Marital Trust and Patriarchy

Marital relationships were said to come under strain when women had different fertility desires than their husbands, leading to them taking contraceptives in secret.

Sometimes contraceptives can cause a bad relationship if a woman goes secretly. I will be upset, she is not married to the doctor, she is married to me. Why does she go to the doctor without telling me? Before she goes for FP she should tell me.

M2 FGD (18–30)

Men disapproved of women taking contraceptives in secret, as it undermined Samburu patriarchy and a husband’s family desires.

If he wants more children and she is taking contraceptives then it causes problems. When a wife tries to tell this to her husband, he does not approve, so she takes them secretly. This causes divorces. The husband will find a wife who will provide him with children.

SSI-03 (18–30)

#### 3.3.5. Role of Women

Thanks to education, the role of women was discussed as having changed considerably compared to the past. While they still hold significant domestic responsibilities in Samburu culture, women now support their families through the running of small businesses.

In the past women stayed at home and carried out work in the manyatta. But now they are more educated, and some of them have started businesses. Their roles have changed because you now find women providing.

SSI-02 (18–30)

Elders perceived that if a woman has several children to look after, then she is unable to provide for her family through these new enterprises.

Now women are providing, they have businesses. But if she has many small children, it will prevent her from doing these things. So we should allow women to use contraceptives.

M2 FGD (45+)

#### 3.3.6. Diversifying Income Streams

Women’s economic empowerment is part of the diversification of income streams that men welcomed. A shift to smaller families through FP was discussed as contributing to this new perspective.

It is good to mix businesses with livestock. When the livestock are away with the Morans, then you can provide for yourself and medicine for the animals.

M1 FGD (18–30)

## 4. Discussion

Bremner et al. argue that there has been an overly simplistic portrayal of the interrelationships between poverty, population growth, and environmental degradation, and that context must be considered to understand what the barriers and facilitators are to successful and community acceptable PHE programmes [31].

All men interviewed in this study were supportive of the environmentally sensitised approach of CHAT’s FP programme, and they wished that they visited more often. In agreement with other literature on PHE programmes [1,3,6], relating family size to resource availability is a compelling strategy to increase FP uptake considering the dependence that the Samburu have on key resources such as water and trees. Along with provision of contraceptives, the educational aspect of the programme fits well with the participants’ universal support for education in their communities.

Importantly, following the African Development and Health Research Centre’s (ADHRC) (2016) recommendations for increased sensitisation of Samburu men to FP in order to improve uptake, CHATs PHE programme presents an example of how this can be achieved. This sensitisation is particularly important in patriarchal Samburu families, where men were against their wives using contraceptives without their approval, seeing themselves as the key decision makers on family size.

All men outlined that the Samburu traditional pastoral livelihood is entirely dependent on natural resources, and almost universally acknowledged the environmental and livelihood benefits of having smaller families. Eight of the nine elders older than 45 (of whom six had more than five children), highlighted that because today ‘life is hard’ it is difficult to provide for large families. A key finding was that although most men ultimately favoured large families, they believed that a compromise of smaller family size should be made in the current context of limited natural resource availability; their increasingly degraded environment could not sustain large families as it had in the past. This reveals that the fertility ideals of Samburu men might be more dynamic than previous cultural assumptions. They understood that having fewer children reduced pressure on parents and ultimately allowed them to support their families with fewer livestock which they could better care for during periods of drought. Having many children and being dependent on large herds of livestock was known to make families more vulnerable during drought, since with few other options, livestock mortality majorly affects the ability of parents to ensure the health and survival of their children.

While the younger age groups of men age 18–30 and 30–45 regularly supported the rationale of smaller families and the benefits they bring, traditional aspirations for large families were still present to some degree in these cohorts. Interestingly, while voicing this opinion of compromise for the Samburu in general, some participants did not intend to practice FP in their own home. Permeating the entire age-cohort, but particularly strong among the elders, the desire to have many children was closely tied to the idea that survival of the Samburu people is dependent on pastoralism and that the land was unfit for any other use. These findings agree with Kaye-Zwiebel and King who found varying perceptions among the Samburu on the adequacy of their grazing land, the economic sufficiency of livestock, and the benefits of conservation [32]. Men described the division of labour within large families for livestock management as a rational decision in the Samburu context. This mirrors Mavanza and Grossman’s findings in Tanzania that were related to fishing rather than pastoral labour [33]: they found that people desired large families to help catch fish, which were their most important source of income and food. As highlighted by eight individuals, the traditional Samburu association of large families and livestock herds with wealth and pride remained a compelling reason to have many children regardless of environmental conditions. This rationale remains a challenge for PHE initiatives, as it is difficult to elicit change in Samburu family desires while men view expansive livestock management as their most fundamental tradition and aspiration. While most participants age 45+ did support family planning to some extent, it is worth considering that these individuals had already fathered multiple children which may have influenced these supportive views. The fact that the younger generation were largely in support of family planning despite (in most cases) not having had any children is an important consideration and may reflect a different perspective on family size compared to elders in the community. Further study is necessary to measure whether fertility ideals such as these among the younger age cohort lead to any changes in family planning outcomes in the near future.

Men also aspired to protect their environment for economic reasons. Through the sale of natural resources and livestock, the environment provides income for the Samburu. Despite occasionally creating conflict through restricting access to traditional grazing land, wildlife conservation was stated to have created economic benefits for the community through employment and tourism, along with the provision of grants for children’s education. However, while large family groups migrate with numerous livestock, conflict with wildlife undermines any economic benefits from conservation. These findings support those of Kitzanides’ (2010) study: when men understand the economic and social value that a healthy environment brings to the community when it is not degraded from overpopulation, they become more supportive of FP [10].

In line with Canning and Schultz’s Bangladeshi study [34], smaller family size and access to education was seen to allow women to take on economic enterprises outside their traditional domestic role. Ahmed et al. argued that FP could reduce MMR [35]; although this particular assertion is impossible to verify in this qualitative study, men described how maternal death had reduced after access to FP, and that greater birth spacing made women visibly healthier. Men also thought that child health had improved, since mothers did not have to feed and care for several young children at once. The new income streams provided by healthy women that start up small businesses marks an evolution of Samburu family dynamics. However, men in all age-groups saw themselves as the key decision makers on family size, a belief which was said to lead to marital problems when women chose to act on their different fertility desires and take contraceptives against their husband’s wishes. Therefore, despite an indication that Samburu women are becoming more empowered to make their own fertility decisions, a husband’s approval may still play a crucial role in determining whether a couple has a smaller family.

Faced with both environmental and economic pressures, men indicated the tribes’ transition towards a more fragmented livelihood, combining traditional livestock management with income generated from conservation, employment following attainment of education, and the aforementioned development of businesses by women. This supports research which argues that the Samburu were transitioning to a more diversified economic strategy including agro-pastoralism, mixed rangeland and wildlife conservation, and urban migration for salaried-labour in order to cope in an increasingly market based economy [32,36,37] Because family size is closely linked with all of these factors (children are often required for livestock management, large families may lead to wildlife conflict (due to greater probabilty of contact), education is more attainable for children of smaller families, and women can enter into the economy if they are not burdened with several children), most men recognised that having smaller families was an appropriate strategy. This supports Kidanu et al.’s Ethiopian findings, where communities encouraged the use of FP because having fewer children, although against traditional aspirations, was a more sustainable strategy in their economic and environmental context [38].

Because CHAT’s PHE initiative is uniquely sensitised to the Samburu livelihood, it plays an important role in educating the community about the relationship between family size and the environment in a region that is experiencing frequent environmental stress. A challenge remains in reaching the remote family groups in Samburu who have not yet been reached by the PHE programme. Men described these communities as holding on to traditional desires for large families and substantial herds of livestock, driving environmental degradation through a resource exhaustion-migration cycle.

Since they are seen as major beneficiaries of this intervention, future research would benefit from qualitative studies that included Samburu women.

This study only included men that had already been exposed to CHAT’s programme, and it remains possible that men developed their given opinions irrespective of the programme or that their thoughts do not translate into any action on family planning uptake. Therefore, to evaluate the impact of CHAT’s PHE programme on FP, maternal health and environmental health, quantitative studies that compare key indicators between PHE and non-PHE control groups that have not been exposed to CHATs programme would be of benefit.

### Limitations

Despite conscientious and detailed Maa to English interpretation by my research assistant, some cultural nuances may have been lost during transcription of the FGDs. Furthermore, due to time and resource constraints, we were not able to verify the accuracy of transcripts with another Maa-speaker.

Although the lead researcher made it clear that he was working independently, the fact that two FPCORPs who work for CHAT accompanied me during data collection created the possibility of social desirability bias: men could have given responses that they believed would please CHAT and lead the mobile clinic to visit more frequently. However, upon reflection on the data collected, we do not believe this to be the case, since men gave a range of rich individual opinions related to family size and the environment, most, but not all of which, supported family planning. This suggested that men felt able to express their views relatively openly. Furthermore, without accompaniment by the trusted FPCORPs, access to the community would not have been granted.

Since the sample size in this study was small and the men included were only those who have been exposed to CHAT’s PHE programme, findings cannot be generalized to communities not exposed to the FP programme. However, they do give an indication as to how men would potentially receive it should expansion continue. Furthermore, the exploratory nature of this study was to investigate and understand perceptions among Samburu men on the links between family size, family planning, and the environment; it will stand as a precursor for further expanded research in the region that includes a larger sample size and a more experimental design.

The lead researcher’s interpretations were regularly discussed with my research assistant to develop both complementary and divergent understandings of the data collected. The researcher was attentive to discrepant cases, where men offered opinions that were considerably different to the other responses. The researcher endeavoured to remain as objective as possible when designing the topic guide and conducting SSIs and FGDs with Samburu men, but the lead researcher cannot discount supportive predispositions that he may have towards CHAT’s PHE programme.

## 5. Conclusions

Because this study lacked a control group who had not been exposed to the PHE intervention, results cannot be objectively extrapolated to the wider Samburu community. However, it can serve as a useful indication as to how men may respond should they participate in similar PHE programmes in the future. This study shows that the unique PHE approach, which relates to the Samburu tribes’ close relationship with the natural environment, may be used as a tool to improve acceptance of FP among men in the Samburu community. The majority of participants highlighted that large families and herds of livestock can lead to wildlife loss, environmental degradation, and unsustainable natural resource use. Because of this, and in combination with frequent and prolonged drought (which some believed was being driven by deforestation by migrating family groups), men found it increasingly difficult to provide for their families. Because of these current circumstances, most men agreed that a compromise of smaller families should be made. However, despite voicing agreement towards reductions in family size in order to ensure sustainable use of natural resources, a small proportion of men did not intend to practice FP in their own homes. This finding highlights the challenges that remain for PHE programmes in translating men’s verbal support for FP into practice.

Economics is a fundamental reason behind recognition of the benefits of smaller families. Men understood their environment to have monetary value, either through conservation for wildlife tourism and related educational grants, or in the maintenance of livestock value. This finding supports previous studies that revealed economic incentives as an important driver of support for FP among men. The economic empowerment and improved health of women that were not burdened with many children was discussed as a major benefit of FP. These economic benefits of FP along with the environmental pressures highlighted by men may reflect a shift towards a more fragmented Samburu livelihood that is less dependent on pastoralism than in the past.

CHAT should continue to expand its mobile clinic and PHE approach, ensuring equitable distribution of services that will ultimately benefit human and environmental health. Furthermore, due to the access to and support from men delivered by the programme, Kenyan FP policy should consider integrating community-based PHE strategies among underserved pastoral groups living in fragile ecosystems.

## Figures and Tables

**Table 1:** Participant demographics.

Age range	18–30	30–45	45+
Number of participants	9	9	9
Marital status			
Single	6	0	0
Married	3	9	9
Number of wives			
0	6	0	0
1	3	6	2
2+	0	3	7
Number of children			
0	6	0	0
1 to 2	3	1	1
3 to 4	0	5	2
5 or more	0	3	6
Education level			
Incomplete primary	0	0	4
Complete primary	0	2	5
Incompete secondary	2	4	0
Complete secondary	7	3	0
Tertiary	0	0	0

## Supplementary Materials

The following are available online at www.mdpi.com/1660-4601/14/5/528/s1. Topic guide.
