# Supplementary material for: Family Planning and the Samburu: A Qualitative Study Exploring the Thoughts of Men on a Population Health and Environment Programme in Rural Kenya
Source: Int J Environ Res Public Health. 2017 May 13;14(5):528. doi: 10.3390/ijerph14050528 (PMC5451979; doi:10.3390/ijerph14050528)
Supplement: Supplementary file 1 [file ijerph-14-00528-s001.pdf]

Topic Guide

Introduction

Greet participant(s). Thank them for their time.
Introduce researcher and research assistant/interpreter.

We are here to listen to and understand your opinions on Family planning and in particular the family planning program carried out by CHAT which links family size to the environment.

Please be aware that this discussion is being audio recorded. Once the recorded information has been written down the recording shall be deleted. Your information such as name shall be made anonymous.

This research is voluntary; you are free to stop taking part at any time.

There are no right or wrong answers, please feel free to speak about anything you believe to be important that does not come up in our questions.

A copy of this research will be made available to you via the Community Health Africa Trust (CHAT).

TOPICS

1. Participant(s) background and livelihood.

- Please tell me about your background and your livelihood/your role in the community.
- How long you have been living in Lodungokwe?
- In what ways has any part of your life changed since you were young?

2. Thoughts/beliefs about family, gender, family size and FP.

- Could you tell us about your family? How many siblings do you have? How old?
- How many wives do you have?
- What is your role in your family?
- What are your beliefs about family? What family desires do you have for the future?
- What is the ideal family size for you, and why is this size important?
- What are the benefits of large families? Do you see any benefits of smaller families?

- Why is family important in your opinion?
- Do you have any different opinions to the Samburu community on family?
- What are your opinions on FP and the use of contraceptives? What is your experience of FP? Do an of your wives use contraceptives? Do you approve of them?
- What is the role of men in the Samburu? What is the role of women in the Samburu? How have these changed in your lifetime?
- How does FP change your relationship with women in your family and community?

3. Environmental and resource thoughts/concerns.

- What is your relationship with the environment? Which resources are most important for your way of life.
- In what ways has your environment and resource availability changed in your lifetime? For example more or less drought/rain?
- How have you coped with environmental stress?
- Has your livelihood changed because of environmental change or resource availability?

4. Thoughts/knowledge on link between family size and environmental resources.

- Have you any thoughts on how having a large family affects your environment and availability of resources? What is your role in your family?
- What are your opinions on your relationship with wildlife in Samburu?
- Do you see any benefits of conservation?
- What is your opinion of the community conservancy movement of the Northern Rangelands Trust? Do you receive benefits from these conservancies?
- Do you have any conflicts with wildlife?
- How does having large families affect the relationship with wildlife and conservation?
- How is the availability of environmental resources such as water or grazing land affecting your attitudes towards family size?

5. Perception of PHE FP program.

- What are your opinions of CHATs FP program?
- How does CHATs program, which discusses family size related to the environment and conservation affect your attitude towards FP and family size?
- How does relating family size to the environment
- How does CHATs program affect your relationship with women in your family?
- Can you see benefits for women in your family and community from this program?

- Are there any parts of the program that you are not happy?
- Which parts of the program are you happy with?
- Do you have any suggestions as to how the program could be improved?

Thank participant(s) for their time. Ask them if there is anything else that they wish to discuss.
